# Obesity accelerates endothelial-to-mesenchymal transition in adipose tissues of mice and humans

**DOI:** 10.3389/fcvm.2023.1264479

**Published:** 2023-09-19

**Authors:** Nicholas W. Chavkin, Tanvi Vippa, Changhee Jung, Stephanie McDonnell, Karen K. Hirschi, Noyan Gokce, Kenneth Walsh

**Affiliations:** ^1^ Robert M. Berne Cardiovascular Research Center, University of Virginia School of Medicine, Charlottesville, VA, United States; ^2^ Department of Cell Biology, University of Virginia School of Medicine, Charlottesville, VA, United States; ^3^ Department of Medicine, Yale Cardiovascular Research Center, Yale University School of Medicine, New Haven, CT, United States; ^4^ Department of Medicine and Whitaker Cardiovascular Institute, Boston University School of Medicine, Boston, MA, United States; ^5^ Hematovascular Biology Center, University of Virginia School of Medicine, Charlottesville, VA, United States

**Keywords:** endothelial-to-mesenchymal transition, obesity, adipose, endothelium, aging, vascular biology

## Abstract

**Introduction:**

Vascular dysfunction and chronic inflammation are characteristics of obesity-induced adipose tissue dysfunction. Proinflammatory cytokines can drive an endothelial-to-mesenchymal transition (EndoMT), where endothelial cells undergo a phenotypic switch to mesenchymal-like cells that are pro-inflammatory and pro-fibrotic. In this study, we sought to determine whether obesity can promote EndoMT in adipose tissue.

**Methods:**

Mice in which endothelial cells are lineage-traced with eYFP were fed a high-fat/high-sucrose (HF/HS) or Control diet for 13, 26, and 52 weeks, and EndoMT was assessed in adipose tissue depots as percentage of CD45^−^CD31^−^Acta2^+^ mesenchymal-like cells that were eYFP ^+^. EndoMT was also assessed in human adipose endothelial cells through cell culture assays and by the analysis of single cell RNA sequencing datasets obtained from the visceral adipose tissues of obese individuals.

**Results:**

Quantification by flow cytometry showed that mice fed a HF/HS diet display a time-dependent increase in EndoMT over Control diet in subcutaneous adipose tissue (+3.0%, +2.6-fold at 13 weeks; +10.6%, +3.2-fold at 26 weeks; +11.8%, +2.9-fold at 52 weeks) and visceral adipose tissue (+5.5%, +2.3-fold at 13 weeks; +20.7%, +4.3-fold at 26 weeks; +25.7%, +4.8-fold at 52 weeks). Transcriptomic analysis revealed that EndoMT cells in visceral adipose tissue have enriched expression of genes associated with inflammatory and TGFβ signaling pathways. Human adipose-derived microvascular endothelial cells cultured with TGF-β1, IFN-γ, and TNF-α exhibited a similar upregulation of EndoMT markers and induction of inflammatory response pathways. Analysis of single cell RNA sequencing datasets from visceral adipose tissue of obese patients revealed a nascent EndoMT sub-cluster of endothelial cells with reduced *PECAM1* and increased *ACTA2* expression, which was also enriched for inflammatory signaling genes and other genes associated with EndoMT.

**Discussion:**

These experimental and clinical findings show that chronic obesity can accelerate EndoMT in adipose tissue. We speculate that EndoMT is a feature of adipose tissue dysfunction that contributes to local inflammation and the systemic metabolic effects of obesity..

## Introduction

An estimated 39%–49% of the world population is overweight or obese (1, 2), and this condition significantly contributes to cardiovascular morbidity and mortality through the systemic dysregulation of inflammatory cytokines (3). Chronic obesity promotes dysfunctional adipose tissue remodeling, resulting in decreased circulation of anti-inflammatory adipokines and increased pro-inflammatory adipokines (3, 4). In obesity, dysfunctional adipose tissue is characterized by macrophage infiltration (5, 6) and polarization towards a pro-inflammatory phenotype (7), necrotic adipocytes surrounded by macrophages that can be visualized as “crown-like” structures (8), tissue fibrosis (9), and vascular rarefaction (10). In this condition, the local pro-inflammatory milieu of the adipose tissue affects adipocytes, fibroblasts, immune cells, and endothelial cells, contributing to adipose tissue dysfunction (3, 11–13). Additionally, adipose tissue hypoxia caused by vascular rarefaction and impaired endothelial cell function will lead to greater cellular stress and inflammation (14–16). These features contribute to a feedforward loop that increasingly favors systemic metabolic dysfunction under conditions of chronic obesity. Thus, a more detailed understanding the multicellular responses that promote dysregulated remodeling in adipose tissue is critical to determining underlying mechanisms of obesity-induced pathologies.

A known consequence of pro-inflammatory cytokines on endothelial cells is a transition from an endothelial to a mesenchymal phenotype (17). This process, termed Endothelial-to-Mesenchymal Transition (EndoMT), has been identified in numerous cardiovascular disease states, including atherosclerosis (18), myocardial infarction (19), pulmonary hypertension (20), and other conditions (21, 22). EndoMT occurring in obese adipose tissue could contribute to the pro-inflammatory, pro-fibrotic, and hypoxic environment that drives dysfunctional adipose tissue remodeling. Notably, a prior clinical study identified transitioning endothelial cells in visceral adipose tissue of obese patients through colocalized immunofluorescence imaging of Smooth Muscle alpha-Actin (mesenchymal marker) and CD31 (endothelial marker) in vessels (23). However, quantitative analysis of the extent of EndoMT enabled by mouse lineage tracing models, and elucidation of the conditions that promote EndoMT in adipose tissue, have not been investigated previously. As EndoMT involves the transition of endothelial cells to a new phenotypic state, studies describing EndoMT can be facilitated by murine transgenic methodologies and by mechanistic and single cell transcriptome analyses of EndoMT in human cells and tissues, respectively.

Herein, we examine EndoMT and underlying mechanisms in adipose tissue with experimental models and clinical datasets. First, employing methods that trace the endothelial cell lineage in mice, it was found that diet-induced obesity accelerates the rate of EndoMT in adipose tissue, particularly in visceral compared to subcutaneous adipose tissue. Transcriptomic analysis of the EndoMT cells from obese mice revealed the upregulation of EndoMT-related genes, including the enrichment of inflammatory response genes. A similar transcriptional profile was also observed in human primary adipose-derived endothelial cells treated with a pro-inflammatory cytokine cocktail to induce EndoMT. Finally, the analysis of single cell RNA sequencing (scRNAseq) datasets of stromal cells from the visceral adipose tissue of individuals with obesity revealed a subset of endothelial cells expressing transcripts associated with EndoMT. The combined analysis of these human single cell datasets identified transcription profiles that were also found in the experimental models of EndoMT. Collectively, these data support the conclusion that obesity accelerates EndoMT in adipose tissue and that this is associated with an inflammatory transcriptional response within the EndoMT cells.

## Materials and methods

### Animal work

All experiments and handling of mice was approved by the University of Virginia Animal Care and Use Committee (ACUC). Mice used in these experiments were generated from two transgenic alleles: Cdh5-CreERT2 (24) and ROSA26-eYFP (25). Tamoxifen was administered to mice at 6 weeks of age by 10 injections of 100 μl of 10 mg/ml Tamoxifen (Sigma, Cat #T5648) dissolved in peanut oil. Injections were performed once per day for 5 days, followed by 2 days of recovery, then once per day for 5 days. Mice were then given a recovery period of 14 days before further assays were performed. Euthanasia of mice was performed by overdose of inhaled isoflurane followed by surgical pneumothorax induction. Both male and female mice were used in these experiments.

### High fat and high sucrose (HF/HS) diet-induced obesity model

Mice were fed *ad libitum* with either a normal chow (NC) diet or high fat and high sucrose (HF/HS) diet (F1850, Bio-Serv) for 13, 26, or 52 weeks starting at 10 weeks after the tamoxifen injection protocol. Several mice were maintained on NC diet for 104 weeks. Total number of mice analyzed at 13 weeks was 8 NC diet (3 female, 5 male) and 15 HF/HS diet (8 female, 7 male), at 26 weeks was 9 NC diet (4 female, 5 male) and 17 HF/HS diet (9 female, 8 male), at 52 weeks was 9 NC diet (4 female, 5 male) and 13 HF/HS diet (8 female, 5 male), and at 104 weeks was 11 NC diet (4 female, 7 male). The composition of the HF/HS diet is 35.8% fat, 36.8% carbohydrate, and 20.3% protein. Mice were weighed at initiation of diet and at euthanasia. Metabolic function in some mice was assessed by glucose tolerance test (GTT) and insulin tolerance test (ITT) before euthanasia. GTT was performed by fasting mice overnight, administering an intraperitoneal injection of 1 mg glucose (50% dextrose injection, Hospira, Cat# 0409-6648-02) per 1 g body weight, then measuring blood glucose by glucose test strips (Accuchek, Roche) at 0, 15, 30, 60, 90, and 120 min after injection. ITT was performed by fasting mice for 4–6 h, administering an intraperitoneal injection of 0.75 U insulin (Humulin, Eli Lilly, Cat# 0002-8215-17) per 1 kg body weight, then measuring blood glucose at 0, 15, 30, 60, 90, and 120 min after injection.

### Tissue collection and analysis

Mice were euthanized according to protocols approved by the University of Virginia ACUC at 13, 26, and 52 weeks after diet, along with several mice at 104 weeks after normal control diet. Subcutaneous adipose tissue was collected from the inguinal fat pad. Visceral adipose tissue was collected from the epididymal fat pad. Lung, kidney, and liver were isolated. Small tissue samples were collected and fixed with 4% paraformaldehyde for immunofluorescent staining and imaging analysis. Cells were isolated from fresh tissues by physical mincing with dissection scissors and then incubation in digestion solution of 5 mg/ml Liberase™ (Sigma, Cat #LIBTM-RO) and 5 μl/ml elastase (Sigma, Cat #E1250) in HBSS (Gibco, Cat #14025092) at 37°C for 60 min. Digested tissue was then centrifuged at 1000 × g for 5 min, and the supernatant containing adipocytes was removed. The cell pellet was resuspended and passed through a 70 μm filter. If used for flow cytometry, cells were fixed with 4% paraformaldehyde for 10 min on ice. Cells were then washed and incubated in immunofluorescent antibodies for flow cytometry analysis and FACS in staining buffer containing 1% BSA (Sigma, Cat #A6003) in PBS (Gibco, Cat #10010023). If used for FACS, a viability stain was included to eliminate cells with ruptured membranes. Immunofluorescent antibodies were used for flow cytometry and FACS: BV421-conjugated anti-mouse CD31 (MEC 13.3, BD Biosciences, Cat #562939), PerCP-conjugated anti-mouse CD45 (30-F11, BD Biosciences, Cat #557235), AlexaFluor594-conjugated anti-Acta2 (D4K9N, Cell Signaling Technologies, Cat #36110). Flow cytometry and FACS were performed using a FACSMelody (BD Biosciences). Flow cytometry was analyzed using FlowJo v10.6.1. Linear and multivariate regression analysis was performed using the Fitting Linear Models function in R with the “lm()” function.

### Fluorescence immunostaining

Adipose tissue was isolated and fixed as described above. Tissues were dehydrated by sequential incubation in ethanol and xylene, embedded in paraffin wax, sectioned to 10 μm thick, incubated on slides to attach, and rehydrated by reverse incubation in xylene and ethanol into PBS. Tissue sections were prepared for immunostaining by performing antigen retrieval using antigen unmasking solution (Vector Labs, Cat #H-3300), permeabilized by incubating with 1% Tween20 in PBS for 30 min at room temperature, then blocked using 3% donkey serum and 0.1% Tween20 in PBS for 1 h at room temperature. Tissue sections were immunostained by incubation in primary antibodies (goat anti-CD31, Q08481, R&D Systems, Cat #AF3628; AlexaFluor594-conjugated rabbit anti-Acta2, D4K9N, Cell Signaling Technologies, Cat #36110; chicken anti-GFP, AbCam, Cat #ab13970) for 1 h at room temperature. After washing, fluorescence-conjugated secondary antibodies were incubated for 1 h at room temperature. DAPI (Thermo Scientific, Cat #62248) was incubated on tissue sections for 5 min at room temperature. Immunostained sections were imaged using a Leica Thunder DMi8 Fluorescence Microscope.

### Bulk RNA sequencing

Bulk RNA sequencing was performed on cells isolated from visceral adipose tissue after FACS or cultured human primary adipose-derived microvascular endothelial cells (described below). RNA from visceral adipose tissue was isolated from mice on NC diet (*n* = 3 male) or HF/HS diet (*n* = 3 male) for 52 weeks. RNA from cultured cells was isolated from Control treatment (*n* = 3) or EndoMT treatment (*n* = 3) as described below. RNA was purified using the RNeasy MinElute kit (Qiagen, Cat #74204). Purified RNA was submitted to the Yale Center for genome analysis for quality control analysis, low-input RNA sequencing amplification, PolyA RNA library preparation, and paired-end 100 bp sequencing (NovaSeq v2, Illumina). Samples were multiplexed in sequencing runs to average 20M reads per sample. Raw sequencing reads were demultiplexed into sample specific Fastq files, which were analyzed by aligning to reference genomes (mouse mm10 or human hg38) by Kallisto v0.46.2 (26). Differential gene expression analysis was performed with Sleuth v0.30.0 (27). Gene set enrichment analysis was performed with GSEA v4.1 software (Broad Institute).

### Cell culture EndoMT model

The EndoMT cell culture model was performed as previously described (23). Briefly, primary human adipose-derived microvascular endothelial cells (HAMVEC, ScienCell, Cat #7200) were obtained and passaged in EGM-2 (Lonza, Cat #CC-3162). For bulk RNA sequencing, HAMVEC were seeded into 6-well plates at 2.5 × 10^5^ cells per well and grown overnight, then induced with either control media (EGM-2) or EndoMT induction media (EGM-2 with 5 ng/ml TNF-*α*, 5 ng/ml IFN*γ*, and 5 ng/ml TGFβ1) for 4 days with an induction media change at 2 days, and RNA was collected and purified using the RNeasy MinElute kit (Qiagen, Cat #74204). Purified RNA was submitted for bulk RNA sequencing and analyzed, as described above. For immunofluorescence staining, HAMVEC were seeded onto 4-well chamber slides (ThermoFisher, Cat #154526) at 2.5 × 10^4^ cells per chamber and subjected to the same EndoMT induction assay as above, then fixed with 4% paraformaldehyde and immunostained in staining buffer containing PBS with 3% donkey serum and 0.1% Tween20 with primary antibodies (Armenian hamster anti-CD31, 2H8, ThermoFisher, Cat #MA3105; AlexaFluor594-conjugated rabbit anti-Acta2, D4K9N, Cell Signaling Technology, Cat #36110), followed by staining with secondary fluorescent-labeled antibodies. DAPI was used as a nuclei counterstain. Immunostained fluorescent cells were imaged using a Leica SP8 Confocal Microscope.

### Analysis of single cell RNA sequencing datasets

Clinical single cell RNA sequencing datasets were obtained from previously published studies (28–30). De-multiplexed, aligned, and normalized data tables were downloaded from online repositories. Seurat v4 (31) was used to analyze each dataset individually. Data was selected for samples from visceral adipose tissue of obese patients, yielding 12,899 cells with 18,306 genes in the Vijay et al. dataset (28), 18,261 cells with 33,538 genes in the Hildreth et al. dataset (29), and 46,539 cells with 31,533 genes in the Emont et al. dataset (30). Cells were scaled and clustered using standard Seurat functions and variables, and UMAP dimensionality reduction was performed with 20 principal components. Endothelial cell clusters yielded 472 endothelial cells with 18,306 genes in the Vijay et al. dataset (28), 1,274 endothelial cells with 33,538 genes in the Hildreth et al. dataset (29), and 1,269 endothelial cells with 33,538 genes in the Emont et al. dataset (30), and they were further analyzed by scaling, clustering, and performing UMAP dimensionality reduction. Endothelial cell clusters were combined by SCT Integration following the Seurat analysis pipeline. Cell scoring was performed by the AddModuleScore function in the Seurat package using described gene sets. Imputation of gene expression was performed using the MAGIC algorithm (32) and visualized over UMAP reduction plots.

### Statistical analysis

Statistical tests were performed as stated in the figure legends. Tests were performed using either GraphPad Prism v9.4.1 or statistical testing from software used in R on either linear regression analysis, multivariate regression analysis, gene set enrichment analysis, or differential gene expression in Sleuth for bulk RNA sequencing analysis.

## Results

### Diet-induced obesity promotes EndoMT in murine adipose tissue

**Figure 1:**
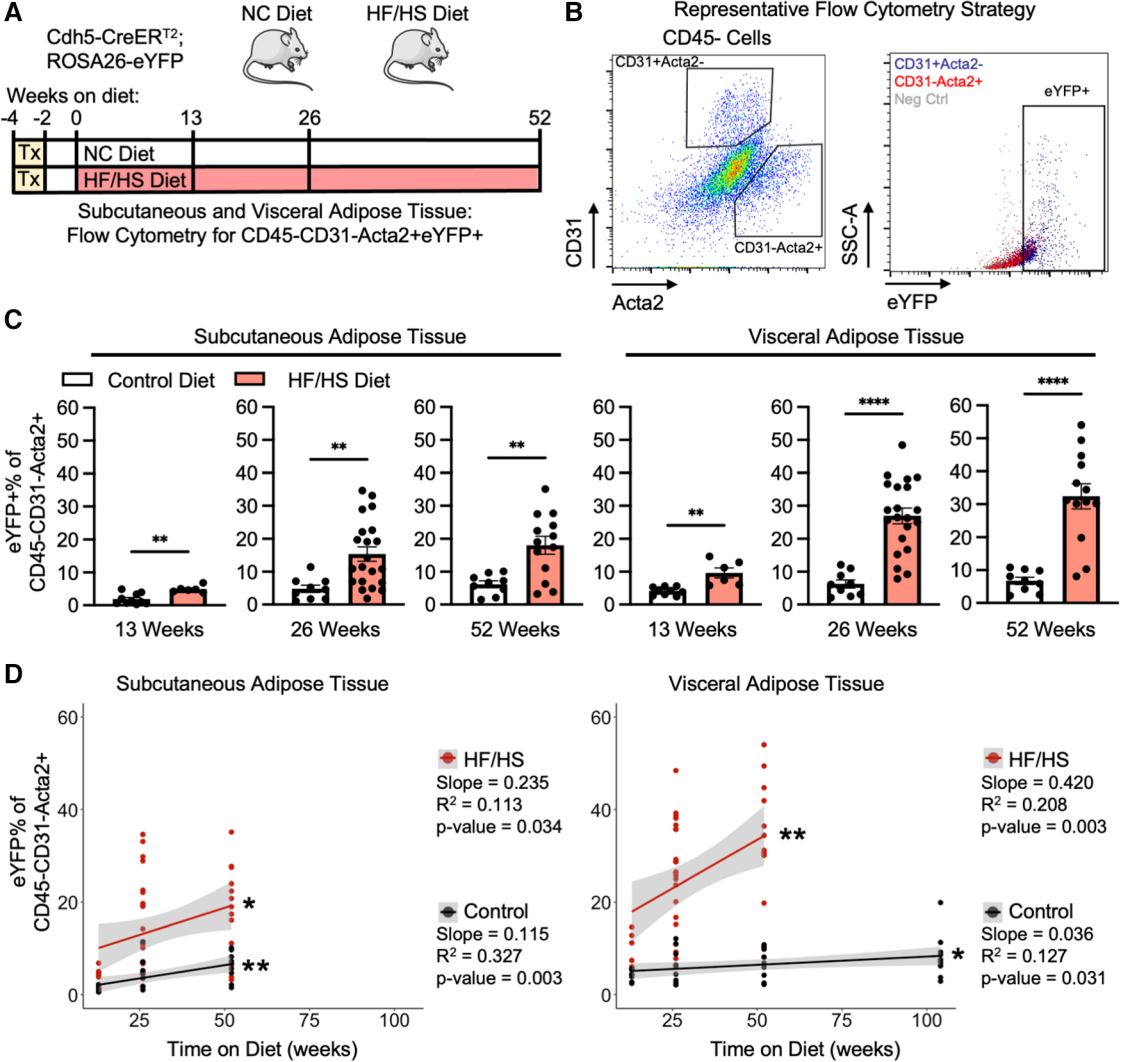
Adipose tissue endoMT in obesity-induced endothelial cell lineage tracing mice. (**A**) Overview schematic of the experimental strategy. (**B**) Representative flow cytometry gating strategy describes the identification of CD31-Acta2 + cells, then quantification of eYFP% of this population. (**C**) EndoMT rate defined as eYFP% of CD45-CD31-Acta2 + cells in subcutaneous and visceral adipose tissue from mice on Control or HF/HS diet for 13, 26, or 52 weeks (student's *t*-test, *p*-values: * <0.05, ** <0.01, *** <0.001, **** <0.0001). (**D**) Linear regression between rate of EndoMT and age in individual mice in subcutaneous or visceral adipose tissue between Control diet and HF/HS diet (linear correlation analysis, *p*-values: * <0.05, ** <0.01).

Endothelial cells lose the expression of endothelial-specific genes as they transition into a mesenchymal phenotype. Therefore, rigorous quantification of EndoMT can be facilitated by the pre-labeling of endothelial cells with a lineage tracing mouse model (Cdh5-CreER^T2^; ROSA26-eYFP). In this system, endothelial cells are pre-labelled with eYFP by tamoxifen-induced Cre recombination, which removes a flox-stop-flox DNA cassette before the eYFP transgene and allows for permanent eYFP expression in any Cdh5 + endothelial cell prior to the initiation of the experimental diets and continues after tamoxifen administration is withdrawn and Cre is inactive. Through this method, endothelial cells that transition away from an endothelial cell phenotype over time and with diet, and therefore lose endothelial cell gene expression, can still be identified by eYFP expression. Mice are then fed either a normal control diet (NC diet) or an obesogenic high fat high sucrose diet (HF/HS diet) for 13, 26, or 52 weeks (Figure 1A). As expected, HF/HS diet significantly increased mouse body mass at all time points, and these mice increasingly exhibited impairments in their metabolic responses to the administration of glucose or insulin, indicative of systemic metabolic dysfunction (Supplementary Figure S1). EndoMT was then quantified in subcutaneous and visceral adipose tissue depots by isolating the stromal vascular fraction and performing flow cytometry to determine the percentage of eYFP^+^ endothelial-derived cells within the CD45^−^CD31^−^Acta2^+^ mesenchymal population (Figure 1B). Quantification showed that HF/HS diet induces a time-dependent increase in EndoMT cells that was greater in the visceral adipose tissue (9.6 ± 1.5% eYFP^+^ of the CD45^−^CD31^−^Acta2^+^ cells at 13 weeks, 27.0 ± 2.4% at 26 weeks, 32.4 ± 3.8% at 52 weeks, mean ± S.E.M) compared to subcutaneous adipose tissue (4.9 ± 0.4% at 13 weeks, 15.4 ± 2.2% at 26 weeks, 18.0 ± 2.7% at 52 weeks, mean ± S.E.M) at all time points analyzed (Figure 1C). In mice on NC diet, a time-dependent increase in EndoMT was also observed in the visceral adipose tissue (4.2 ± 0.4% at 13 weeks, 6.3 ± 1.2% at 26 weeks, 6.7 ± 1.1% at 52 weeks, mean ± S.E.M) and subcutaneous adipose tissues (1.9 ± 0.5% at 13 weeks, 4.8 ± 1.1% at 26 weeks, 6.2 ± 1.0% at 52 weeks, mean ± S.E.M) (Figure 1C). The rate of EndoMT in HF/HS diet compared to NC diet increased over time on diet in visceral adipose tissue (+5.5% eYFP^+^ of the CD45^−^CD31^−^Acta2^+^ cells, +2.3-fold at 13 weeks; + 20.7%, +4.3-fold at 26 weeks; +25.7%, +4.8-fold at 52 weeks) and subcutaneous adipose tissue (+3.0%, +2.6-fold at 13 weeks; +10.6%, +3.2-fold at 26 weeks; +11.8%, +2.9-fold at 52 weeks). Further, the percentage of EndoMT in subcutaneous and visceral adipose tissue are also linearly correlated with time on diet, with a greater correlation slope in mice on HF/HS diet in both adipose depots (0.235% per week in subcutaneous, 0.420% per week in visceral) compared to NC diet (0.115% per week in subcutaneous, 0.036% per week in visceral) and a greater slope of linear regression in HF/HS visceral adipose tissue compared to HF/HS subcutaneous adipose tissue (Figure 1D). Of note, EndoMT percentage was significantly correlated with age of mice on NC diet. This was investigated by maintaining mice on NC diet for 104 weeks and quantifying EndoMT percentage in visceral adipose tissue, revealing a continuation of the linear correlation between EndoMT percentage and age.

**Figure 2:**
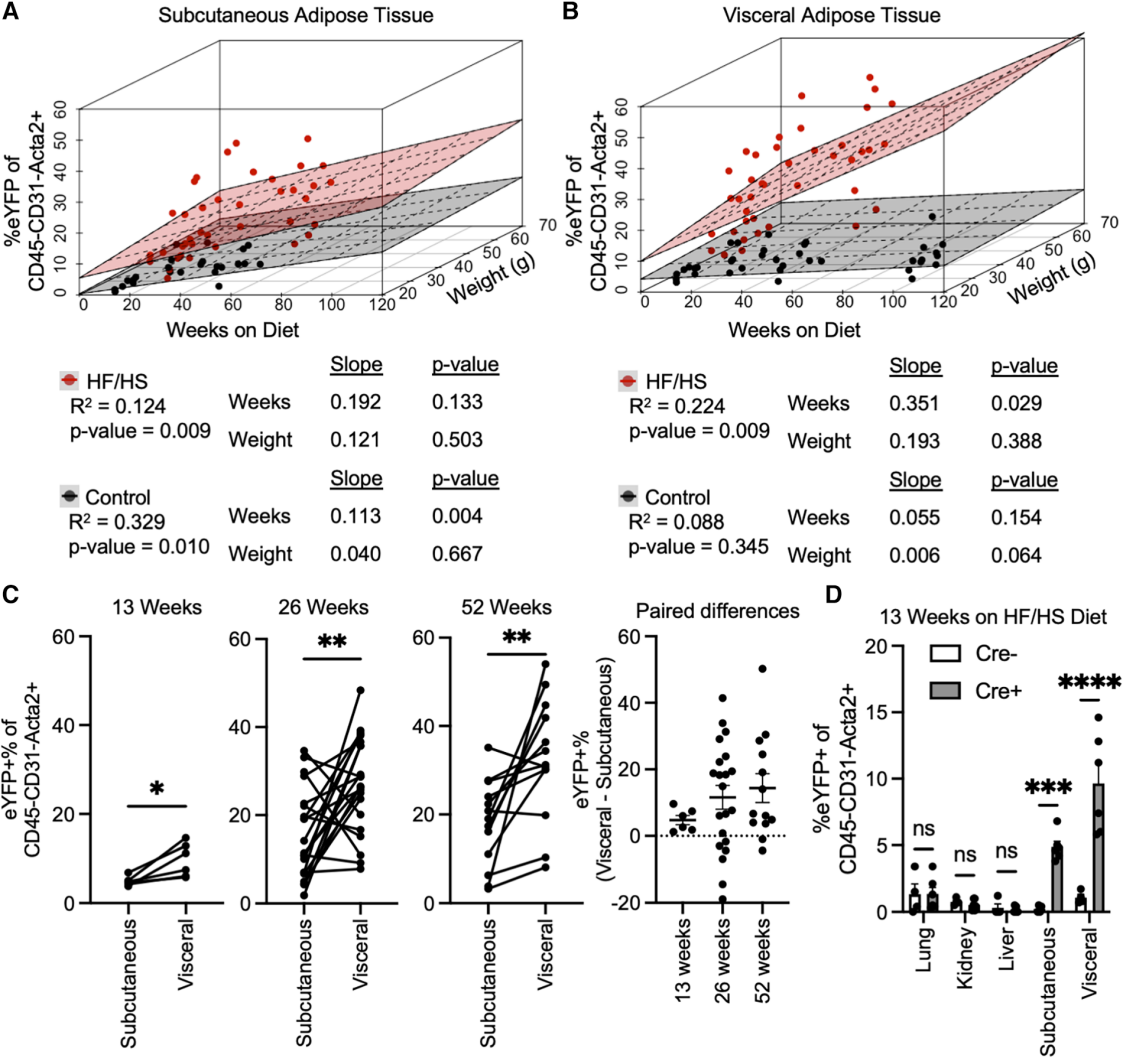
Associations of EndoMT to time on diet, body weight, and adipose tissue location. Multivariate regression analysis quantified relative association of EndoMT to weeks on diet or weight of mice in (**A**) subcutaneous or (**B**) visceral adipose tissue (slope and *p*-value presented from multivariate regression analysis). (**C**) Pairwise analysis of EndoMT in subcutaneous and visceral adipose tissues from the same mice are connected by lines in 13, 26, and 15 weeks on HF/HS diet, with paired differences quantified and presented as EndoMT in visceral minus EndoMT in subcutaneous adipose tissue (paired student's *t*-test, *p*-values: * <0.05, ** <0.01). (**D**) Rate of EndoMT was quantified in lung, kidney, liver, subcutaneous adipose tissue, and visceral adipose tissue in negative control mice (Cre- with no lineage tracing) and in reporter mice (Cre + with lineage tracing) at 13 weeks on HF/HS diet (one-way ANOVA with post-hoc Tukey, *p*-values: * <0.05, ** <0.01, *** <0.001, **** <0.0001).

As many variables could be contributing to the extent of EndoMT in adipose tissue, the association of EndoMT with time on diet, body weight, and sex were investigated by multivariate regression analyses. These analyses showed that time on diet has a greater contribution to rate of EndoMT accumulation compared to overall mouse body weight in either subcutaneous or visceral adipose tissue (Figures 2A,B). This relationship was found regardless of whether mice are on HF/HS diet or NC diet. As expected, the relative rates of EndoMT accumulation are greater in visceral adipose compared to subcutaneous tissue depots, and this was also evident through paired longitudinal analyses of EndoMT in subcutaneous and visceral adipose tissues of individual mice at 13, 26, and 52 weeks on diet (Figure 2C). Sex as a biological variable for EndoMT was also investigated. Although overall weight gain is higher in male mice on HF/HS diet, the rate of EndoMT accumulation did not significantly differ between male and female mice in subcutaneous or visceral adipose tissue at 13, 26, or 52 weeks on either NC diet or HF/HS diet (Supplementary Figure S2). Finally, the extent of EndoMT was also investigated in other tissues. At 13 weeks on HF/HS diet, little or no EndoMT could be detected in lung, kidney, or liver, as determined by comparison with Cre-negative control mice, whereas EndoMT was readily observed in the subcutaneous and visceral adipose tissue (Figure 2D). Together, these analyses show that time on diet and visceral location provide the greatest contributions to the development of EndoMT in adipose tissue.

### Adipose EndoMT cells display increased inflammatory and TGFβ transcriptional responses

**Figure 3:**
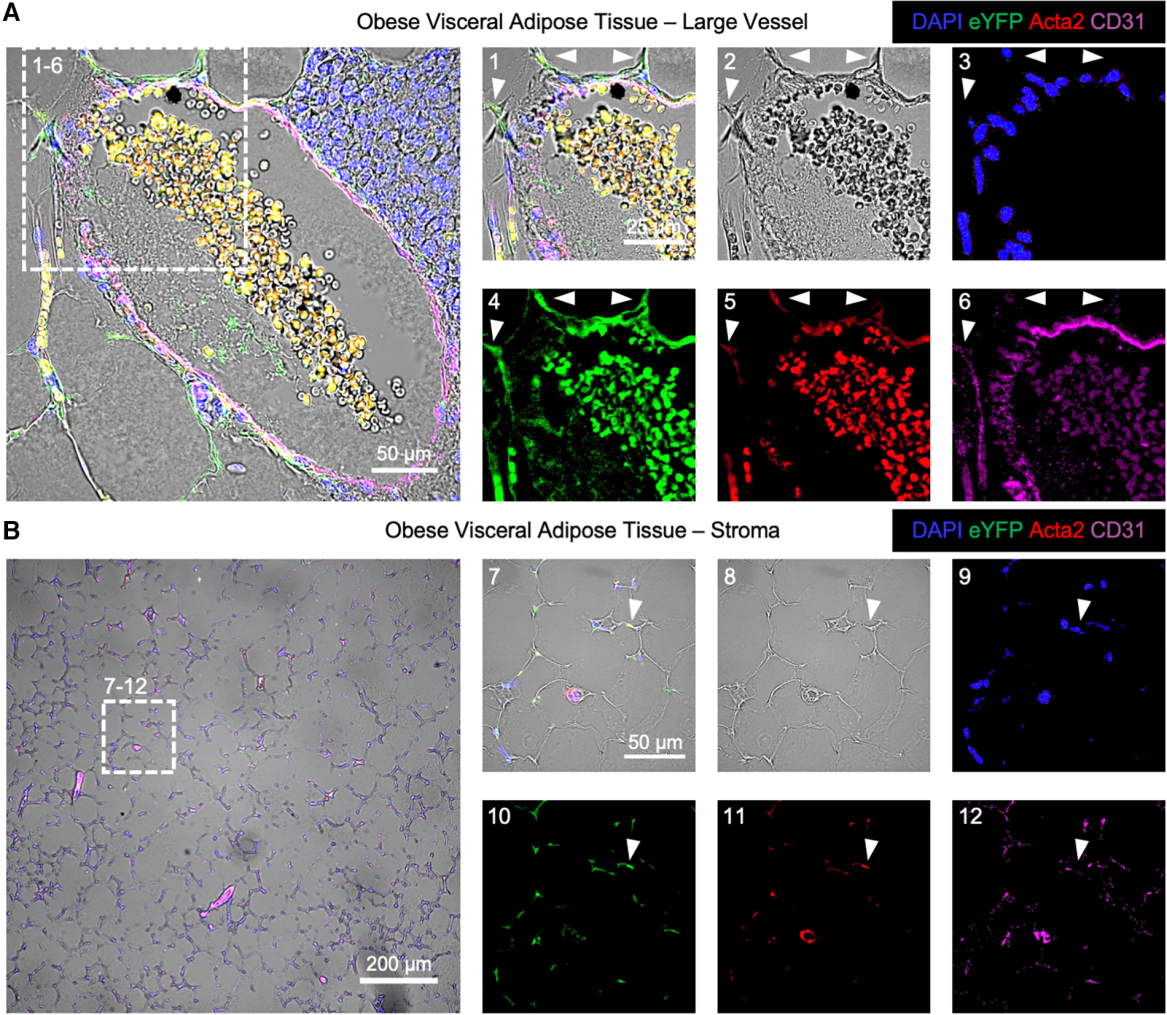
Immunofluorescence imaging of endoMT cells in obese visceral adipose tissue of mice. Visceral adipose tissue from Cdh5CreER^T2^; Rosa26eYFP lineage tracing mice on HF/HS diet was immunofluorescently labeled for cell nuclei by DAPI, the eYFP reporter, Acta2, and CD31 overlaid with brightfield imaging to visualize EndoMT cells as DAPI + eYFP + Acta2 + CD31-. (**A**) A representative large vessel is shown, with a magnified section to highlight EndoMT cells by white arrows in (1) all channels, (2) brightfield, (3) DAPI, (4) eYFP, (5) Acta2, and (6) CD31. (**B**) A representative image of the stroma with small vessels is shown, with a magnified section to highlight an EndoMT cell by a white arrow in (1) all channels, (2) brightfield, (3) DAPI, (4) eYFP, (5) Acta2, and (6) CD31.

The localization of EndoMT cells was performed in the endothelial lineage tracing model through immunofluorescent imaging. In this analysis, a cell that is positive for eYFP and Acta2 but negative for CD31 is scored as an EndoMT-positive cell. Samples of visceral adipose tissue from mice fed HF/HS diet were isolated at 26 weeks on diet, and the tissues were fixed, sectioned, and immunostained for CD31, Acta2, and eYFP. Imaging of these sections revealed that EndoMT cells are found on the exterior to arterial vessels with protrusions that are oriented away from the vessel lumen (Figure 3A). A similar localization pattern has been observed in clinical specimens where the putative transitioning endothelial cells are identified within arterial vessels via the simultaneous expression of CD31 and ACTA2 (18, 20, 22, 23). In addition to the perivascular location, EndoMT cells were also identified in the regions of the visceral adipose tissue stroma (Figure 3B).

**Figure 4:**
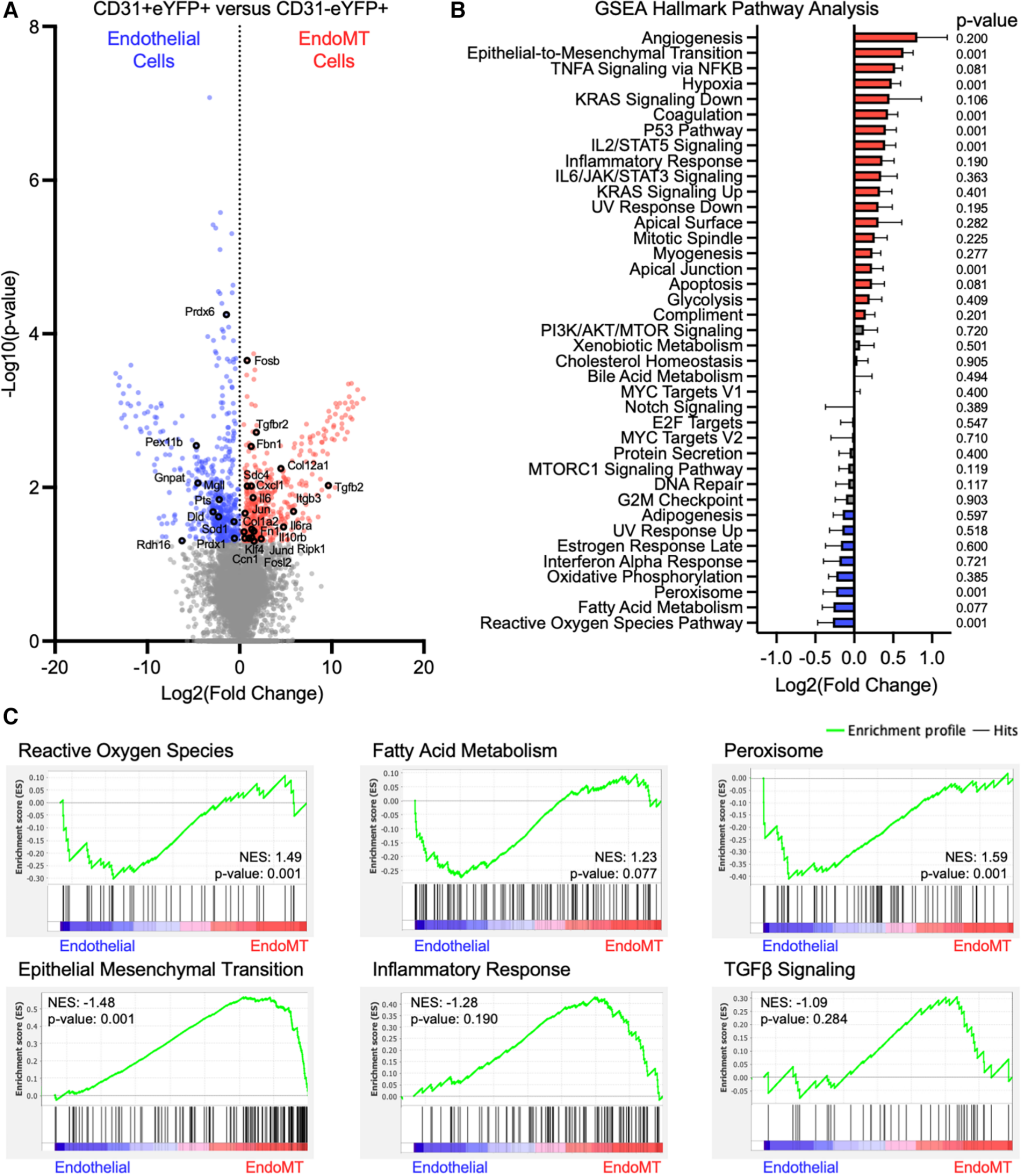
Transcriptomic analysis of murine endoMT cells in visceral adipose tissue of obese mice. (**A**) Differential expression of all genes identified through bulk RNA sequencing of CD31 + eYFP + endothelial cells vs. CD31-eYFP + EndoMT cells (*n* = 3 in each condition) was visualized by volcano plot showing the log2 (fold change) vs. the -log10 (*p*-value) of the change in gene expression, with genes significantly upregulated in endothelial cells in blue, EndoMT cells in red, and specific genes involved in endothelial cell function or EndoMT labeled. (**B**) Enrichment of GSEA Hallmark Pathways in all differentially expressed genes were visualized by log2 (fold change) and *p*-values listed, with pathways upregulated by 10% in red and downregulated by 10% in blue. (**C**) Enrichment profiles presented for terms enriched in endothelial cells (reactive oxygen species, fatty acid metabolism, peroxisome) and in EndoMT cells (epithelial mesenchymal transition, inflammatory response, TGFβ signaling).

The transition from endothelial-to-mesenchymal phenotype corresponds to transcriptional changes that can have functional consequences (33). To investigate these transcriptional changes in the current context, stromal vascular fraction cells were isolated from the visceral adipose tissue of endothelial lineage-traced mice that were fed HF/HS diet for 52 weeks, and cells were separated by FACS into CD31^+ ^eYFP^+^ endothelial cells and CD31^−^eYFP^+^ EndoMT cells. RNA was isolated and bulk RNA sequencing was performed after low-input cDNA amplification on three paired replicate samples. The transcriptional profile of 885 genes was significantly different between endothelial cells and EndoMT cells, with 218 transcripts enriched in endothelial cells and 667 transcripts enriched in EndoMT cells (Figure 4A). Several genes enriched in endothelial cells are involved in reactive oxygen species signaling (e.g., *Sod1*, *Prdx1*), fatty acid metabolism (e.g., *Prdx6*, *Prdx1*, *Mgll*, *Pts*, *Dld*, *Rdh16*), and peroxisome signaling (e.g., *Pex11b*, *Gnpat*). Several genes enriched in EndoMT cells are involved in TGFβ signaling (e.g., *Tgfbr2*, *Tgfb2*), inflammation (e.g., *Il6*, *Il6ra, Il10rb, Fosb, Jun, Fosl2, Klf4, Cxcl1, Ripk1*), epithelial-to-mesenchymal transition (e.g., *Fbn1*, *Itgb3*, *Ccn1*), and extracellular matrix components (e.g., *Fn1*, *Col1a2*, *Col12a1*) (Figure 4A). Gene set enrichment analysis (GSEA) revealed that pathways characteristic of EndoMT cells, including epithelial-to-mesenchymal transition, inflammatory response, and TGFβ signaling, were significantly enriched or trending towards enrichment in CD31^−^eYFP^+^ EndoMT fraction (Figures 4B,C). In contrast, GSEA of the CD31^+ ^eYFP^+^ endothelial cells revealed a significant enrichment in reactive oxygen species signaling and peroxisome biology, and trending towards enrichment in fatty acid metabolism, which are hallmark pathways of vascular endothelial cells (Figures 4B,C). The cumulative transcriptional pattern of EndoMT cells compared to endothelial cells, including upregulation of genes known to promote inflammation and enrichment of downstream inflammatory pathways, suggests an activation of inflammatory processes during obesity associated EndoMT in adipose tissue.

### Human adipose endothelial cells can be induced to undergo EndoMT in cell culture

**Figure 5:**
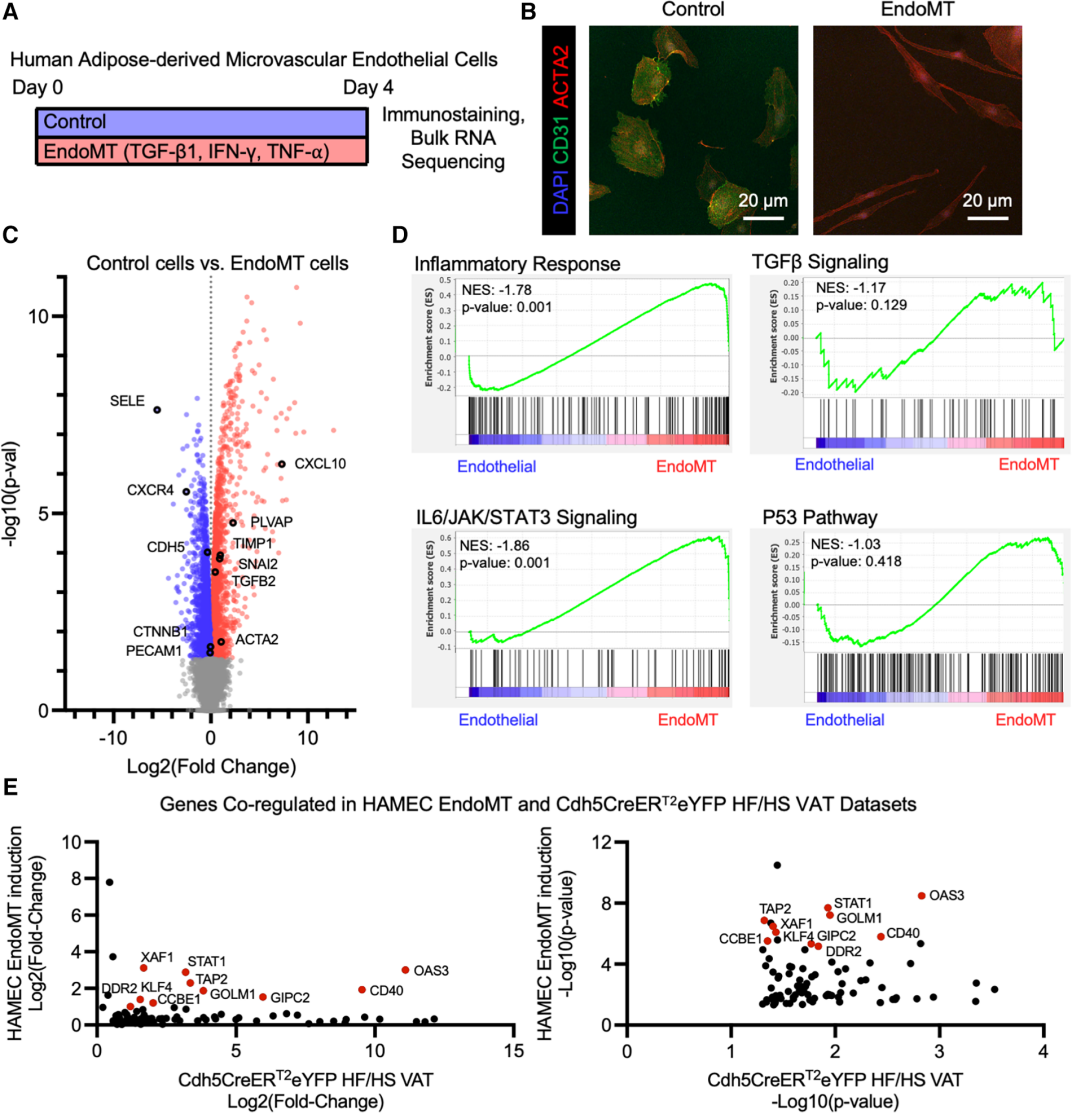
Endomt in cultured human adipose-derived microvascular endothelial cells. (**A**) Overview schematic of experimental strategy. (**B**) Representative immunofluorescence image of HAMEC induced with pro-inflammatory cytokines, visualizing DAPI, CD31, and ACTA2. (**C**) Differential expression of all genes identified through bulk RNA sequencing of Control vs. EndoMT cells (*n* = 3 in each condition) was visualized by volcano plot showing the log2 (fold change) vs. the -log10 (*p*-value) of the change in gene expression, with genes significantly upregulated in Control cells in blue, EndoMT cells in red, and specific genes involved in endothelial cell function or EndoMT labeled. (**D**) Enrichment plots of GSEA Hallmark Pathway analysis of inflammatory response, TGFβ signaling, IL6/JAK/STAT3 signaling, and P53 pathway. (**E**) Genes that are significantly upregulated in both EndoMT conditions (HAMEC and Cdh5CreER^T2^; Rosa26eYFP HF/HS datasets), presented as relative log2 (fold change) and -log10 (*p*-value) quantifications, and genes with a 2-fold increase in both conditions labeled and highlighted in red.

The potential for human adipose endothelial cells to undergo EndoMT was also assessed in a cell culture system. Primary human adipose-derived microvascular endothelial cells (HAMEC) were treated with a pro-transition cocktail of TGF-β1, IFN-*γ*, and TNF-*α* to induce EndoMT, as previously described (23) (Figure 5A). The response of HAMEC to the treatment was visualized by fluorescence immunostaining of CD31 and ACTA2, revealing that cells transition from rounded CD31^+^ endothelial cells to spindle-shaped ACTA2^+^ mesenchymal-like cells (Figure 5B). RNA sequence analysis, performed on control and EndoMT cocktail-treated HAMEC, revealed genes that were significantly upregulated and downregulated (Figure 5C). GSEA revealed significant enrichment of genes involved in inflammatory response and IL6/JAK/STAT3 signaling, and trending enrichment of P53 pathway and TGFβ signaling, in the transitioned cells (Figure 5D). Additionally, a comparison of transcriptomes from the obesogenic mouse (Figure 4) and human cell culture EndoMT models identified 86 genes that are significantly upregulated in both RNA sequencing datasets, with 10 genes significantly upregulated greater than 2-fold in both datasets (Figure 5E). These 10 genes are directly involved in the response to inflammatory stimuli (*STAT1, TAP2, CD40, OAS3, XAF1*), extracellular matrix remodeling (*CCBE1, DDR2*), or epithelial-to-mesenchymal transition (*KLF4, FOLM1, GIPC2*). Collectively, these analyses indicate that human adipose endothelial cells have the potential to undergo EndoMT, yielding a transcriptional profile that is similar to the transcriptional profile identified in the murine obesity-induced EndoMT model.

### Individual transitioning EndoMT cells are present in adipose tissues of individuals with obesity

**Figure 6:**
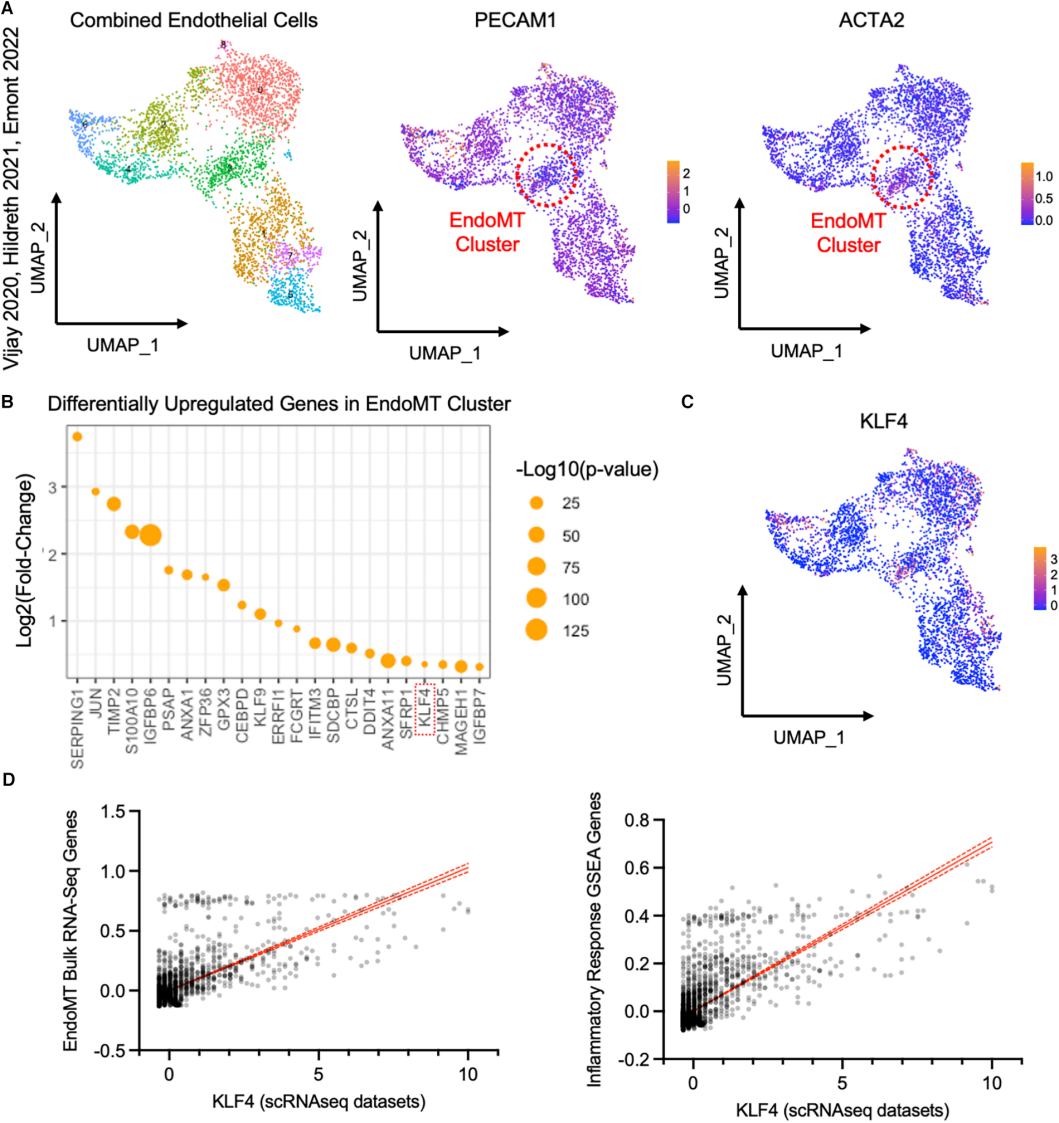
Analysis of endothelial cells from single cell RNA sequencing datasets of visceral adipose tissue from obese patients. (**A**) UMAP of endothelial cells from datasets published in Vijay 2020, Hildreth 2021, and Emont 2022, with an EndoMT cell cluster highlighted by a red circle, identified by reduced relative *PECAM1* expression and increased relative *ACTA2* expression plotted by UMAP. (**B**) Log2 (fold change) and -log10 (*p*-value) of genes upregulated in the EndoMT cell cluster compared to other endothelial cells. (**C**) Relative *KLF4* expression in endothelial cells plotted by UMAP. (**D**) Linear correlation between expression of *KLF4* and combined expression of genes that are co-regulated in bulk RNA sequencing datasets from HAMEC and Cdh5CreER^T2^;Rosa26eYFP (*r*^2^ = 0.456, *p*-value <0.0001, left) and genes in the inflammatory response GSEA (*r*^2^ = 0.527, *p*-value <0.0001, right).

To detect evidence of an EndoMT transcriptional profile in human adipose tissue, we analyzed publicly available scRNAseq datasets from the visceral adipose stromal vascular fraction of patients with obesity (28, 29, 30). Raw single cell RNA expression data from each of these three datasets was visualized by UMAP dimensionality reduction analysis and clustered into similar cell types, with endothelial cell clusters identified by expression of *PECAM1* (Supplementary Figures S4A,B). Within the endothelial cell clusters within each dataset, we could detect a sub-cluster of cells that expresses a relatively low level of *PECAM1* and relatively high level of *ACTA2* (Supplementary Figures S4C,E), suggesting these cells are undergoing EndoMT. Combining and integrating data from the endothelial cell clusters of all three datasets revealed a similar cluster of cells that are *PECAM1^low^* and *ACTA2^high^* in the pooled data (Figure 6A). This *PECAM1^low^ACTA2^high^* cluster displayed an enrichment of genes that was similar to the EndoMT gene set enrichment observed in the obese lineage-traced mice and the HAMEC treated with EndoMT transitioning cocktail (Figure 5E and Supplementary Figure S5A). Inflammatory response genes associated with EndoMT processes in the murine and cell culture models were also observed in the human *PECAM1^low^ACTA2^high^* cluster (Supplementary Figure S5B). Furthermore, there was a correlation between EndoMT transcript expression and inflammatory response gene expression within this cluster of human cells (Supplementary Figure S5C).

Differential gene expression analysis of the pooled EndoMT cluster from human visceral adipose tissue identified the enrichment of 23 genes (Figure 6B). Several of these genes have been associated with EndoMT in vascular-related diseases (*KLF4* (22, 34–36), *TIMP2* (18, 37), *JUN* (38)), and others have been associated with the similar process of Epithelial-to-Mesenchymal transition (*ANXA1* (39), *ERRFI1* (40), *IFITM3* (41), *IGFBP7* (42)). Notably, the upregulation of *KLF4* (Kruppel-Like Factor 4) (Figure 6C) was also observed in the bulk RNA sequencing analyses of the murine adipose tissue and human cell culture models (Figure 5E). KLF4 is a key transcription factor previously shown to promote EndoMT in cell culture models (22, 34, 43, 44). Thus, *KLF4* expression in the individual cells of the pooled EndoMT cluster from human visceral adipose tissue was compared to expression patterns of the gene sets enriched in the bulk RNA sequencing analyses of EndoMT in the model systems from Figures 4, 5. This analysis revealed that increasing *KLF4* expression in human visceral adipose tissue correlates with increasing expression of the EndoMT gene set components identified in the model system. Similarly, a strong correlation was found between *KLF4* expression, and the expression of genes annotated in the Inflammatory Response GSEA pathway (Figure 6D). Collectively, these analyses reveal a consistency of data among the human adipose tissue, human cell culture, and murine lineage-tracing model, supporting the hypothesis that EndoMT occurs in visceral adipose tissue under conditions of obesity.

## Discussion

In this study, we investigated the effects of obesity on the transition of vascular endothelial cells to cells that display a mesenchymal phenotype within adipose tissue. EndoMT in adipose tissue was visualized, quantified and transcriptionally defined using endothelial cell lineage-traced mice fed an obesogenic diet, a cell culture model of HAMEC treated with an EndoMT pro-transition cocktail, and scRNAseq datasets from visceral adipose tissue biopsies of obese patients. In the mouse model, it was found that an obesogenic diet induces EndoMT in adipose tissue in a time-dependent manner. The increase in rate of EndoMT was greater in the visceral compared to subcutaneous adipose tissue depot, and a greater rate of EndoMT was more closely correlated to time-on-diet rather than overall mouse weight. RNA sequence analysis comparing vascular endothelial cells (eYFP^+ ^CD31^high^ACT2A^low^) and EndoMT cells (eYFP^+ ^CD31^low^ACT2A^high^) within the mouse visceral adipose documented the expression of EndoMT pathways that had been identified previously in other tissues, and also identified a robust increase in transcripts that encode for pro-inflammatory proteins. In the human cell culture model, RNA sequence analysis of HAMEC treated with the pro-transition cocktail of TGF-β1, TNF-alpha, and IFN-gamma also displayed transcriptional profiles consistent with a transition to a mesenchymal phenotype and the upregulation of multiple pro-inflammatory pathways. Finally, the interrogation of scRNAseq data sets from the visceral adipose tissues of obese individuals revealed subsets of cells with transcriptional profiles that are consistent with cells that are undergoing EndoMT. These three independent lines of evidence provide consistent evidence for EndoMT in the vasculature of adipose tissue in response to obesogenic stimuli.

These results suggest several mechanisms by which EndoMT could contribute to adipose tissue dysfunction. The transcriptional analyses of samples from murine models and human cell culture, as well as of scRNAseq data from clinical samples, all identified a widespread increase in pro-inflammatory signaling in the EndoMT cells. These data are consistent with a prior clinical study that identified histological markers of EndoMT in adipose tissue of obese patients and provided evidence that EndoMT cells promote tissue dysfunction through the release of pro-inflammatory extracellular vesicles (23). In the current study, we found an induction of IL-6 signaling in EndoMT cells. In other tissues, IL-6 has been shown to be produced by EndoMT cells (45), and that it is associated with EndoMT processes in pulmonary arterial hypertension (46), cardiac valves (47), and fibrosis in hearts and kidneys (48). In addition to inflammation, our transcriptomic analyses of EndoMT also identified enrichment of several pro-fibrotic genes, which has been associated with EndoMT cells in cardiovascular diseases (17). Fibrosis is a hallmark of dysfunctional adipose tissue that inhibits adipocyte lipid accumulation and adipocyte expansion, leading to pro-inflammatory cytokine secretion by adipocytes and impaired adipocyte function that increases systemic insulin resistance and glucose intolerance (49). Endothelial cell dysfunction in adipose tissue can promote fibrosis through impaired angiogenesis and increased inflammatory processes that activate adipose fibroblasts (11, 14) many of which we identified to be enriched in EndoMT cells. Furthermore, EndoMT affects vascular integrity and promotes vascular rarefaction (50, 51), which could further promote adipose tissue dysfunction. Vascular rarefaction promotes hypoxia, and we identified hypoxia as an enriched pathway in EndoMT cells in obese murine adipose tissues. Additionally, vascular rarefaction is induced in adipose tissue by an obesogenic diet (16) and is known to promote tissue fibrosis and dysfunction (52). Consistent with these possible connections to tissue pathology, we found that the rate of EndoMT was greater in visceral adipose tissue compared to subcutaneous adipose tissue. These pathologic responses in the adipose microenvironment (inflammation, fibrosis, vascular rarefaction) have been shown to be greater in visceral adipose tissue compared to subcutaneous adipose tissue, contributing to greater obesity-induced adipose tissue dysfunction in visceral depots (3). Future studies into differences between progression of EndoMT in subcutaneous vs. visceral adipose tissue depots may further elucidate the connection between obesity associated pathology and adipose EndoMT. Through these pathways involving hypoxia, inflammation, and fibrosis, EndoMT may constitute an aspect of endothelial cell dysfunction that contributes to many of the features that are recognized to be components of the pathophysiology of adipose tissue dysfunction.

Our results suggest that aging, in addition to obesity, may be contributing to the EndoMT in adipose tissue. We found that mice on a control diet also displayed detectable EndoMT in adipose tissue that increases with time, albeit at a slower rate. Supporting this notion, we also found that the amount of time mice spent on a HF/HS diet has a greater association with degree of EndoMT than does the weight of the mice. Several studies have suggested that age may induce EndoMT through activation of pathways that are shared between aging and EndoMT processes (33), including oxidative stress and inflammation (53), Nrf2 inhibition (54), and mTOR inhibition (55). Additionally, it has been reported that human endothelial cells undergo EndoMT after replicative aging in culture (56, 57), suggesting that this effect of aging is intrinsic to endothelial cells. It can be speculated that the extrinsic effects of obesogenic diet can accelerate these processes. In this regard, EndoMT is known to be induced by high concentrations of glucose (58, 59) and oxidized low-density lipoprotein (60), and inhibited by high-density lipoproteins (61), indicating that changes in serum metabolites and lipoprotein particles could be features of the obesogenic diet that promotes EndoMT in adipose tissues. Our results showed that an obesogenic diet did not promote EndoMT in lung, liver, or kidney tissues, but was instead restricted to adipose tissue. These findings may suggest that other aspects of early adipose tissue dysfunction induced by an obesogenic diet may initiate or promote adipose EndoMT. It is also possible that obesity could exacerbate EndoMT in other tissues, but this may also require direct injury to these tissues. Further investigations are necessary to test this hypothesis.

Our analysis of the human scRNAseq datasets revealed changes in several transcripts that were unique to the identified EndoMT cluster of visceral adipose tissue. Of these genes, the transcription factor KLF4 was identified in transcript analysis of the cultured human adipose tissue endothelial cell model, and its expression level correlated with the expression of genes associated with inflammation and EndoMT. KLF4 is of particular interest because of its reported role in promoting EndoMT in several vascular diseases, including cerebral cavernous malformations (22, 34), coronary arterial wall damage in Kawasaki disease (35), and tumor vascular dysfunction (36). It has also been shown that the downregulation of *KLF4* in cultured endothelial cells inhibits the process of EndoMT (43, 44). In addition to *KLF4*, several other genes identified in the EndoMT cluster have been associated with EndoMT, including *TIMP2* (18, 37) and *JUN* (38), while other genes have been associated with the similar process of Epithelial-to-Mesenchymal Transition including *ANXA1* (39), *ERRFI1* (40), *IFITM3* (41), *IGFBP7* (42). These gene expression patterns provide further support for the notion that EndoMT in adipose tissue is a feature of obesity in humans and mice. Furthermore, these gene expression patterns also resemble inflammation-induced EndoMT (62), which leads to the development of tissue fibrosis, another feature of adipose tissue dysfunction (49), as well as atherosclerosis and neointimal thickening (63, 64).

Recent studies, in various contexts, have highlighted the limitations of drawing conclusions about EndoMT from individual lines of evidence, as discussed in several recent reviews (33, 65, 66). Thus, we have endeavored to provide multiple lines of corroborating experimental evidence from mouse and human systems to document the presence of EndoMT in adipose tissue. However, we acknowledge that this study has limitations. For example, the murine lineage tracing strategy employed here does not provide evidence for a permanent transition to a cell with a mesenchymal identity. A partial EndoMT or reversible EndoMT process has been described in myocardial infarction (19), and the lineage tracing model does not distinguish between permanent and partial EndoMT. Our methods for identifying endothelial cells may also have limitations, since some tissue-specific endothelial cells may have reduced or no expression of traditional endothelial cell markers that might not be labeled by the Cdh5-Cre^ERT2^; Rosa26-eYFP model or the CD31-mediated flow cytometry strategy [e.g., liver sinusoidal endothelial cells (67)]. Although this may limit our interpretation of results on EndoMT in other tissues, this has not been reported for adipose endothelial cells. Additionally, age-dependent declines in the efficiency of the Cdh5-Cre^ERT2^ construct or stability of Rosa26-eYFP reporter may impact the measured rates of EndoMT. However, these murine reporter-based limitations would decrease, and not increase, the calculated rates of EndoMT. Furthermore, the murine HF/HS diet induces both adiposity and overall metabolic dysfunction. Thus, our model cannot distinguish between fat mass expansion *per se* or the subsequent metabolic dysfunction as the mechanistic cause of the EndoMT in adipose tissue. The HF/HS model is designed to recapitulate clinical consequences of obesity, and our analysis of clinical samples also revealed an EndoMT signature in visceral adipose tissue of obese patients. Thus, we believe these findings have clinical significance for the understanding of obesity-induced adipose tissue dysfunction. Finally, interpretations of cell transitions in scRNAseq data analyses can be influenced by technical limitations associated with cell or nuclei isolations from patient tissue samples or the lack of sensitivity of next-generation sequencing. However, we believe that the identification of multiple unique gene transcripts associated with EndoMT in human adipose tissue strongly supports the interpretation that EndoMT is present in these clinical samples.

In summary, the combination of results from an obesogenic murine lineage tracing model, cell culture induction model, and the scRNAseq datasets from human adipose tissue support the conclusion that obesity promotes EndoMT in adipose tissue.

## Data Availability

The datasets presented in this study can be found in online repositories. The names of the repository/repositories and accession number(s) can be found below: https://www.ncbi.nlm.nih.gov/geo/, GSE241015.
