# Supplementary material for: Obesity accelerates endothelial-to-mesenchymal transition in adipose tissues of mice and humans
Source: Front Cardiovasc Med. 2023 Sep 19;10:1264479. doi: 10.3389/fcvm.2023.1264479 (PMC10546194; doi:10.3389/fcvm.2023.1264479)
Supplement: Supplementary file 1 [file Datasheet1.pdf]

Supplementary Material

Supplementary Figures

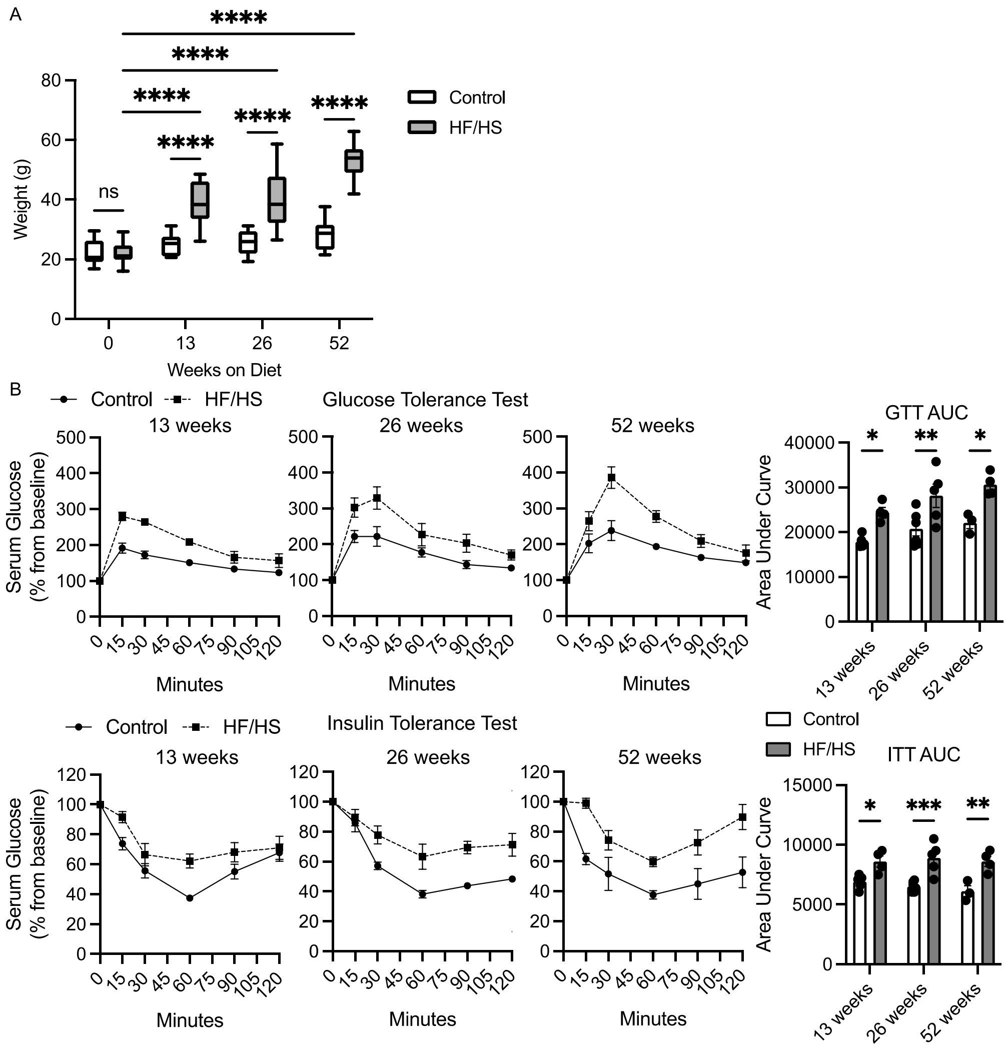

Supplementary Figure 1. Analysis of metabolic parameters in mice. A) Weights of mice on Control or HF/HS diet at 0, 13, 26, and 52 weeks on diet (one-way ANOVA post-hoc Tukey, p-values: * < 0.05, ** < 0.01, *** < 0.001, **** < 0.0001). **B**) Glucose tolerance test results of mice on control or HF/HS diet for 13, 26, and 52 weeks, with area-under-curve reported, and insulin tolerance test results of mice on control or HF/HS diet for 13, 26, and 52 weeks, with area-under-curve reported (student's t-test between each time point, p-values: * < 0.05, ** < 0.01).

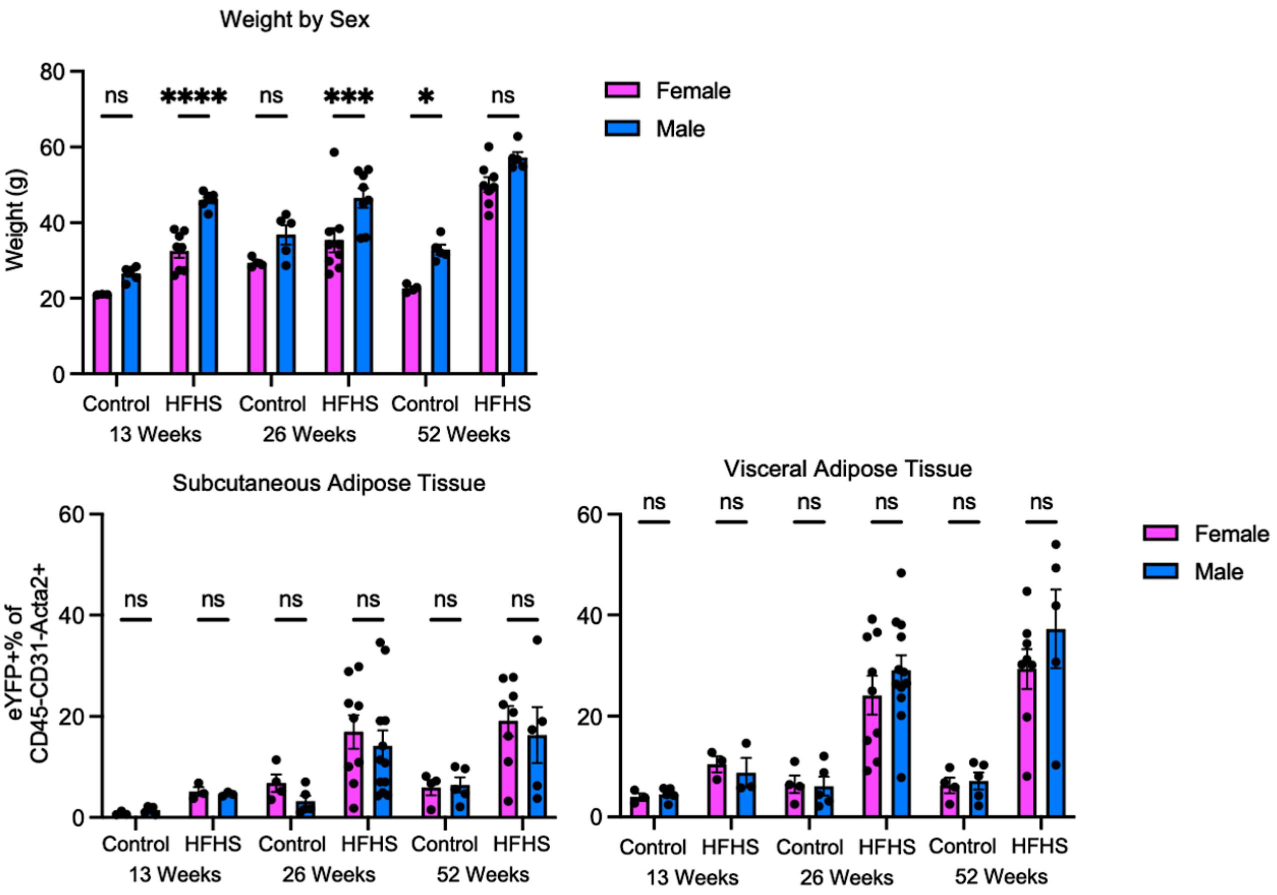

Supplementary Figure 2. Sex as a Biological Variable in EndoMT in Adipose Tissue. Weight, EndoMT in subcutaneous adipose tissue, and EndoMT in visceral adipose tissue compared between Female and Male mice.

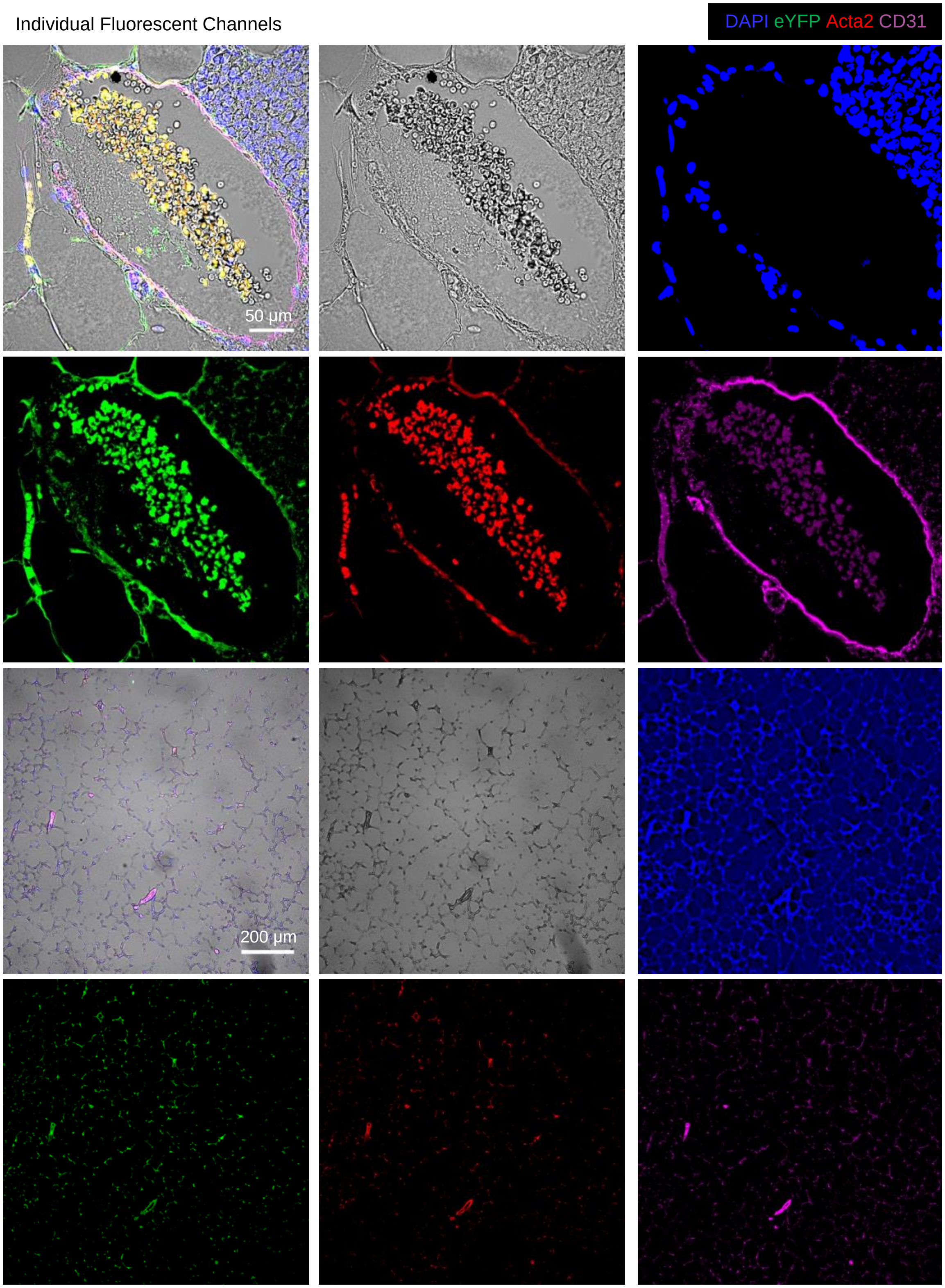

Supplementary Figure 3. Immunofluorescence imaging of EndoMT cells in obese visceral adipose tissue. Individual fluorescence channels of images.

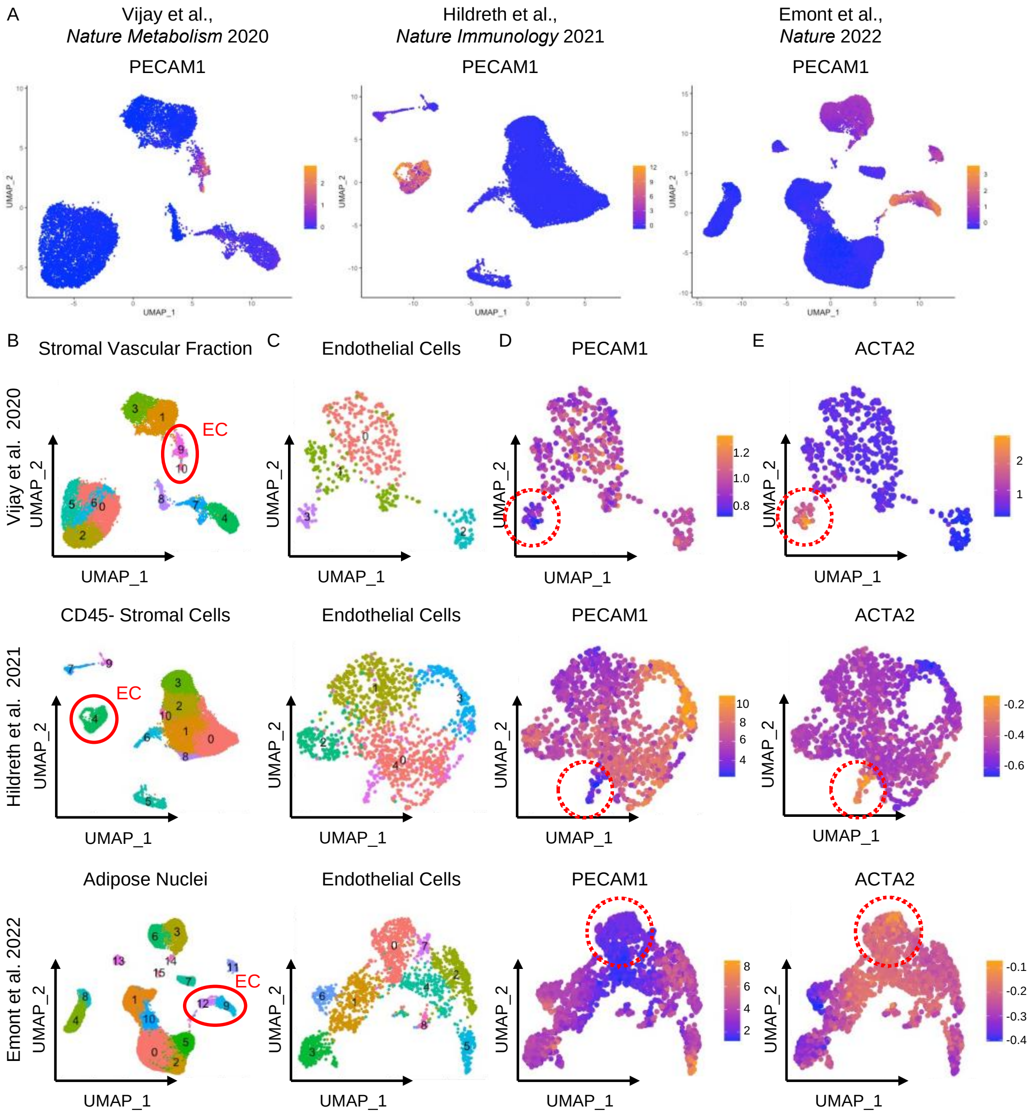

Supplementary Figure 4. Re-analysis of endothelial cells from single cell RNA sequencing datasets of visceral adipose tissue from obese patients. A) UMAP plots of all cells in single cell RNA sequencing datasets in Vijay 2020, Hildreth 2021, and Emont 2022 with expression of PECAM1 highlighting endothelial cell clusters. UMAP plots showing **A**) all cells with endothelial cells highlighted by a red circle and **B**) endothelial cells, with EndoMT cells highlighted with a red dotted circle with **C**) relative *PECAM1* expression (low in EndoMT cells), and **D**) relative *ACTA2* expression (high in EndoMT cells) in each individual dataset.

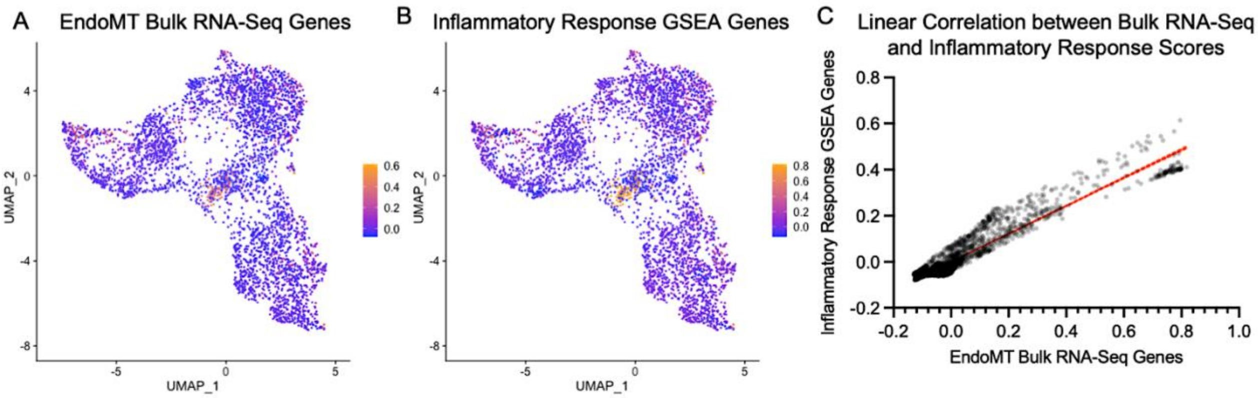

Supplementary Figure 5. Re-analysis of endothelial cells from single cell RNA sequencing datasets of visceral adipose tissue from obese patients. Relative gene set scoring of genes that are co-regulated in **A**) bulk RNA sequencing datasets from HAMEC and Cdh5CreER^{T2};Rosa26eYFP, and **B**) genes in the inflammatory response GSEA. **C**) Linear correlation between Bulk RNA-Seq and Inflammatory Response scores in individual endothelial cells ($r^2 = 0.900$, p-value < 0.0001).
